# Characterization and Fine Mapping of the Stay-Green-Related Spot Leaf Gene *TaSpl1* with Enhanced Stripe Rust and Powdery Mildew Resistance in Wheat

**DOI:** 10.3390/ijms26094002

**Published:** 2025-04-23

**Authors:** Xiaomin Xu, Xin Du, Yanlong Jin, Yanzhen Wang, Zhenyu Wang, Jixin Zhao, Changyou Wang, Xinlun Liu, Chunhuan Chen, Pingchuan Deng, Tingdong Li, Wanquan Ji

**Affiliations:** 1 State Key Laboratory for Crop Stress Resistance and High-Efficiency Production, College of Agronomy, Northwest A&F University, Yangling, Xianyang 712100, China; 18309260341@163.com (X.X.); wzhenyu2018@163.com (Z.W.); zhjx881@163.com (J.Z.); chywang2004@nwsuaf.edu.cn (C.W.); liuxinlun@nwafu.edu.cn (X.L.); chchch8898@163.com (C.C.); dengpingchuan@nwsuaf.edu.cn (P.D.); tingdongli@nwafu.edu.cn (T.L.); 2 Department of Life Sciences and Medicine, University of Science and Technology of China, Hefei 230026, China; duxin2023@ustc.edu.cn; 3 College of Life Sciences, Fudan University, Shanghai 200438, China; jinyanlong@fudan.edu.cn; 4 Center for Agricultural Genetic Resources Research, Shanxi Agricultural University, Taiyuan 030031, China; wangyanzhen9605@163.com

**Keywords:** *TaSpl1*, disease resistance, fine mapping, photosynthesis, stay-green

## Abstract

Lesion mimic phenotypes, characterized by leaf spots formed in the absence of pathogens or pests, are often associated with reactive oxygen species (ROS) accumulation and cell necrosis. This study identified a novel and stable homozygous spotted phenotype (HSP) from the F_8_ population of common wheat (XN509 × N07216). The yellow spots that appeared at the booting stage were light-sensitive, and accompanied by cell necrosis and H_2_O_2_ accumulation. Compared with homozygous normal plants (HNPs), HSPs exhibited enhanced resistance to stripe rust and powdery mildew without compromising yield. RNA-Seq analysis at three stages revealed that differentially expressed genes (DEGs) between HSPs and HNPs were significantly enriched in KEGG pathways related to photosynthesis and photosynthesis-antenna proteins. GO analysis highlighted chloroplast and light stimulus-related down-regulated DEGs. Fine mapping identified *TaSpl1* within a 0.91 Mb interval on chromosome 3DS, flanked by the markers *KASP188* and *KASP229*, using two segregating populations comprising 1117 individuals. The candidate region contained 42 annotated genes, including 14 DEGs based on previous BSR-Seq data. PCR amplification and qRT-PCR verification identified the expression of *TraesCS3D02G022100* was consistent with RNA-Seq data. Gene homology analysis and silencing experiments confirmed that *TraesCS3D02G022100* was associated with stay-green traits. These findings provide new insights into the genetic regulation of lesion mimics, photosynthesis, and disease resistance in wheat.

## 1. Introduction

Leaves are vital for photosynthetic energy capture, a process responsible for over 90% of crop biomass production [1]. Leaf color variations are common in nature, although chloroplast degradation typically accompanies natural leaf senescence [2]. Leaf color mutations, particularly those affecting photosynthetic efficiency, often reduce crop yield. Lesion mimic mutants (LMMs) are a unique category of these mutants, characterized by spontaneous necrotic lesions that resemble pathogen-induced symptoms despite the absence of actual infection [3]. Environmental factors, such as light, humidity [4], temperature, and nutrient availability, also influence the development of these mutants [5]. Although lesion mimic mutants often exhibit premature leaf senescence, they frequently show enhanced disease resistance.

Since the discovery of lesion mimic mutants, they have been extensively studied in various species, including *Arabidopsis* [6], maize [7], barley [8], tomato [9], rice [10], cotton [11], peanut [12], soybean [13], and wheat [14]. However, few such mutations have been mapped or cloned in wheat. Most reported wheat lesion mimics have resulted from chemical mutagenesis rather than spontaneous mutations. For instance, lesion formation in the segregating population from the cross Yanzhan 1/Zaosui 30 was attributed to light-dependent cell death, with genes *lm1* and *lm2* mapped to chromosomes 3BS and 4BL, respectively [15]. Other studies have mapped *lm3*, which forms small, discrete white lesions [16], and *lm4*, a gene flanked by SSR markers with intervals of 0.51 cM and 0.77 cM, which confers improved stripe rust resistance [17]. Additionally, *lm5* on chromosome 2AL increased resistance to both stripe rust and powdery mildew [18], and *lm6* was mapped to chromosome 6BL within a 1.18 Mb region [19].

The hypersensitive response (HR), a programmed cell death mechanism, is frequently linked to the enhanced resistance observed in lesion mimic mutants. Wheat autophagy-related genes *ATG4*, *ATG6*, and *ATG8* are known to regulate resistance to powdery mildew [20]. Moreover, *ATG8* enhances resistance to stripe rust by mediating cell death [21]. Reactive oxygen species (ROS) accumulation, along with increased levels of phytoalexins, phenolic compounds, and callose, triggers a plant HR, thereby fortifying resistance against pathogens [22]. Most rice lesion mimic mutants exhibit enhanced disease resistance [23], as exemplified by the *oshpl3* mutant, which showed increased resistance to bacterial blight [24]. Similarly, *spl40* displayed resistance to 14 out of 16 bacterial blight pathogen races due to upregulated SA and JA signaling genes [25]. In wheat, resistance to powdery mildew was validated in both field and greenhouse conditions for the *lm3* mutant [16], while *lm5* conferred resistance to both stripe rust and powdery mildew [18]. Furthermore, *TaLSD1*, a functional homologue of *AtLSD1*, negatively regulates hypersensitive cell death and contributes to disease resistance against stripe rust in wheat [26]. Lesion mimic mutants, therefore, represent ideal models for studying programmed cell death and plant defense mechanisms.

In this study, we identified a novel, stably inherited spot mutation in wheat. The spot formation, influenced by light and accompanied by cell necrosis and H_2_O_2_ accumulation, impacted photosynthesis-related indicators without adversely affecting yield. RNA-Seq analysis across three developmental stages revealed significant DEGs enriched in photosynthesis-related pathways. Fine mapping identified the spot gene *TaSpl1* within a 0.91 Mb interval on chromosome 3DS. Subsequent qRT-PCR and gene silencing experiments indicated that *TraesCS3D02G022100* plays a key role in stay-green regulation. These findings provide valuable genetic resources for enhancing disease resistance in wheat and contribute to a deeper understanding of lesion mimic formation and photosynthesis regulation.

## 2. Results

### 2.1. Spots Affected by Light Exhibited Cell Necrosis and H_2_O_2_ Accumulation

**Figure 1:**
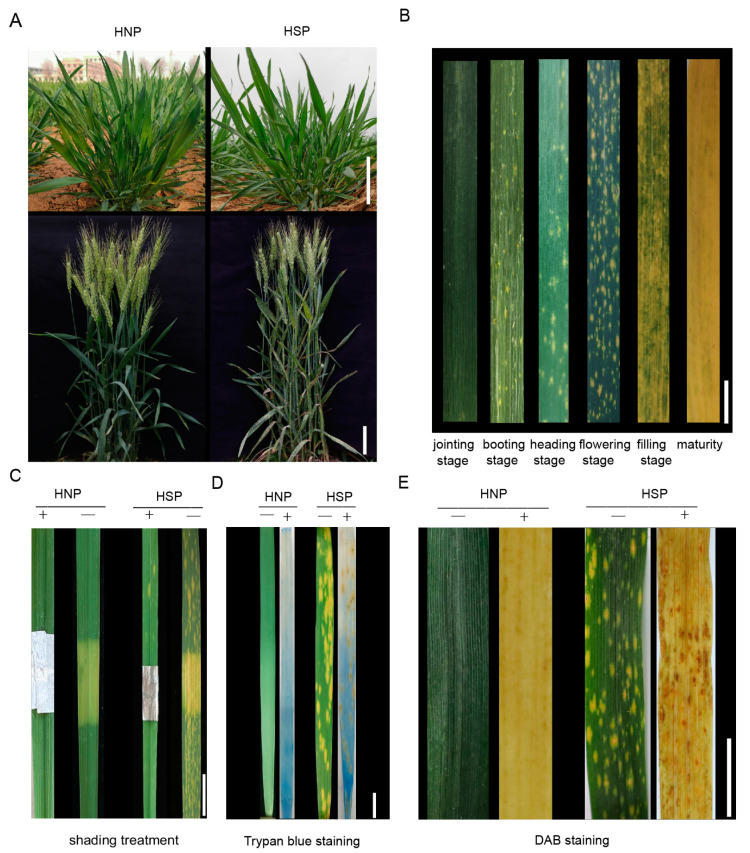
The formation and development of spots. (**A**) The contrast of homozygous normal plant (HNP) and homozygous spotted plant (HSP) at seedling stage (Scale bar, 5 cm) and flowering stage (Scale bar, 10 cm). (**B**) The variation in HSP spot leaves in different growth stages, including jointing stage, booting stage, heading stage, flowering stage, filling stage, maturity. Scale bar, 4 cm. (**C**) The shading leaves comparison of HNP and HSP after 20 days. Scale bar, 2 cm. (+) and (−) indicate before shading and after shading. (**D**) Trypan blue staining of HNP and HSP leaves. Scale bar, 2 cm. (**E**) DAB staining of HNP and HSP leaves. (−) and (+) indicate whether the leaves are treated. Scale bar, 3 cm.

Under normal field conditions, no phenotypic differences were observed between HNP and HSP at the seedling or adult plant stages (Figure 1A). At the booting stage, spots first appeared near the leaf base and gradually spread toward the apex. These yellow, irregular spots expanded until they covered the entire leaf, progressing from the bottom to the top of the plant. As leaves senesced, they turned completely yellow and dry (Figure 1B). In HSP plants, the spots were confined to leaves, while HNP plants maintained green leaves until senescence.

Comparing covered and uncovered leaf areas over 20 days revealed that covered regions developed irregular yellow traces, albeit fewer than in exposed areas. Upon foil removal, the previously covered regions remained nearly white in HNPs, unlike the yellowing or whitened uncovered areas in HSPs (Figure 1C). These observations indicate that light influenced the development of spots but was not the sole factor driving their appearance.

From the onset of spots to leaf death, the lesions exhibited yellow pigmentation without overt necrosis. Trypan blue staining, commonly used to detect necrotic cells in wheat mutants [27], revealed that while HNP leaves became transparent after decolorization with chloral hydrate, HSP leaves exhibited light yellow staining interspersed with small blue spots, indicating cell necrosis (Figure 1D). Early-stage spots were more prone to necrosis, particularly in larger, connected areas.

DAB staining, which detects H_2_O_2_ accumulation, revealed reddish-brown staining in HSP leaf spots, while HNP leaves remained white or transparent (Figure 1E). These results demonstrate that H_2_O_2_ accumulation occurred in HSP spot areas compared to normal leaves.

### 2.2. Spot Formation Enhances Disease Resistance Without Yield Penalties

**Figure 2:**
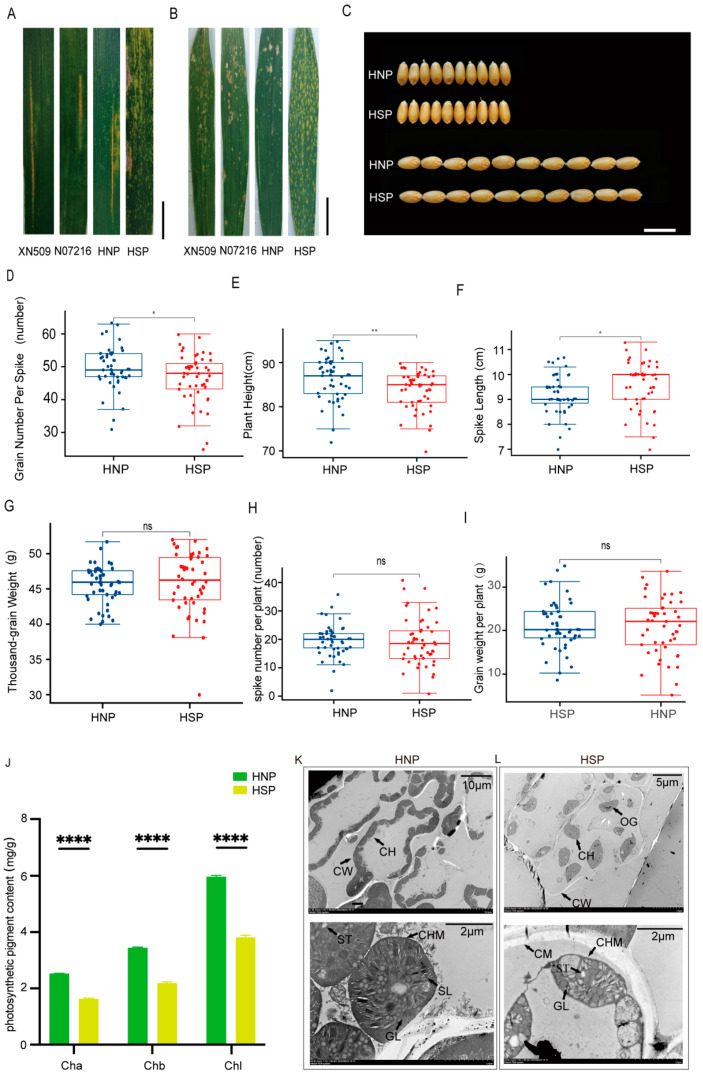
Evaluation of disease resistance, agronomic traits, and photosynthesis-related indicators between HNP and HSP. (**A**) The evaluation of stripe rust resistance for XN509, N07216, HNP, and HSP at field. Scale bar, 3 cm (**B**) The evaluation of powdery mildew resistance for XN509, N07216, HNP, and HSP at field. Scale bar, 4 cm. (**C**) Grain Width (GW) and Grain Length (GL) between HNP and HSP. Scale bar, 1 cm. (**D**) Mean grain number per spike between HNP and HSP. (**E**) Mean plant height between HNP and HSP. (**F**) Mean spike length between HNP and HSP. (**G**) Mean thousand-grain weight between HNP and HSP. (**H**) Mean spike number per plant between HNP and HSP. (**I**) Mean grain weight per plant between HNP and HSP. ns, *p* > 0.05; *, *p* < 0.05; **, *p <* 0.01; ****, *p <* 0.0001. (**J**) The photosynthetic pigment contents of HNP and HSP at flowering stage. Ch a: chlorophyll a; Ch b: chlorophyll b; Chl: chlorophyll, Error bars means + SD. The leaf cell structure and chloroplast structure of HNP (**K**) and HSP. (**L**) CH: chloroplast; CW: cell wall; CM: cytomembrane; CHM: Chloroplast membrane; ST: starch grain; SL: stroma lamella; GL: grana lamella; OG: osmiophilic globule.

When inoculated with mixed races *Pst* CY32 and *Pst* CY34, XN509, N071216, and HNP plants exhibited comparable infection severity. Grades “2” and “3” represent as susceptible. In contrast, HSP plants showed no stripe rust spores, with only necrotic patches along the leaf edges. Grade “0” represents as resistant (Figure 2A, Table 1). In response to *Bgt* E09 inoculation, HNP, XN509, and N071216 leaves displayed extensive spore infections from the bottom to the top of the plant. Infection types were identified as susceptible. However, HSP leaves remained free of spores or lesions. Grade “0” was identified as resistant to disease (Figure 2B, Table 1). These findings suggest that the formation of spots conferred resistance to both stripe rust and powdery mildew.

Agronomic trait analysis revealed no significant differences in GW and GL between HNP and HSP plants (Figure 2C). However, significant differences were observed in the GNS (Figure 2D), PH (Figure 2E), and SL (Figure 2F), with HSP plants having notably longer spike lengths. Yield components, including the TGW (Figure 2G), SNP (Figure 2H), and GWP (Figure 2I), showed no significant differences between the HNP and HSP. These results indicate that while spots influenced certain agronomic traits, they had a minimal impact on overall yield.

### 2.3. Spots Influence on Photosynthesis-Related Indicators in Leaves

Statistical analysis using GraphPad Prism 9.4.1 revealed significant differences in chlorophyll a, chlorophyll b, and total chlorophyll content between HNP and HSP leaves. Chlorophyll levels were significantly lower in HSP plants (*p* < 0.001) (Figure 2J), potentially affecting leaf color and photosynthetic efficiency.

The number and structure of chloroplasts may directly influence chlorophyll content. Transmission electron microscopy revealed intact cell walls and membranes in HNP mesophyll cells, with approximately 20 closely packed, elliptical chloroplasts per cell (Figure 2K, upper). In HSP cells, the cell walls were disrupted, and about 10 oval chloroplasts were sparsely distributed (Figure 2L, upper).

Submicroscopic observations further highlighted structural differences. HNP chloroplasts had intact, smooth edges, well-organized stroma and grana thylakoids, numerous starch granules, and no visible gaps (Figure 2K, lower). In contrast, HSP chloroplasts exhibited irregular edges, scattered and deformed thylakoids, fewer starch granules, and more gaps and voids (Figure 2L, lower).

Comparative analyses of photosynthetic indicators revealed significant differences in Pn and Gs between HNPs and HSPs, but no notable variations in Ci and Tr. Gs values for XN509 were similar to HNPs, while N071216 values closely aligned with HSPs. Notably, HNPs displayed higher WUE, reflecting more efficient photosynthesis relative to transpiration (Table 2). These findings suggest that spots had a negative effect on photosynthesis.

### 2.4. Expression Patterns of DEGs Between HNP and HSP by RNA-Seq

**Figure 3:**
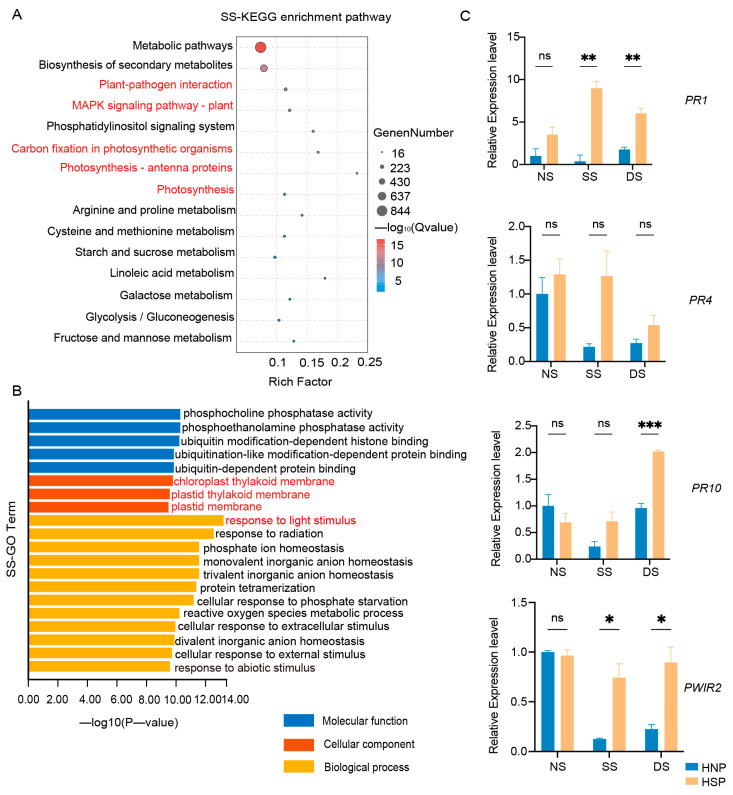
Enrichment analysis of DEGs. (**A**) KEGG enrichment analysis of DEGs in SS stage between HNP and HSP. (**B**) GO enrichment of down-regulated DEGs between HNP and HSP in SS stage. (**C**) Expression of defense-related genes in HNP and HSP. The error bars represent the standard deviation (SD) between biological replicates. NS represents the stage of no spot, SS represents the stage of some spots just appeared and DS represents the stage of spots distributed on the whole leaf. ns, *p* > 0.05; *, *p* < 0.05; **, *p* < 0.01; ***, *p* < 0.001.

A total of 18 samples were subjected to RNA-Seq, generating 535.98 M reads (160.02 Gb) of clean data, with each sample yielding at least 7.93 Gb of clean data (Table S2). The Q30 base percentage across samples exceeded 92.48%. KEGG enrichment analysis revealed significant DEG enrichment during SS and DS stages, notably in photosynthesis, photosynthesis-antenna proteins, and carbon fixation pathways (Figure 3A and Figure S2A). GO enrichment further indicated that down-regulated DEGs were linked to chloroplast components, the chloroplast thylakoid membrane, and responses to light stimulus and reactions in both SS and DS stages (Figure 3B and Figure S2B).

While LMM genes vary extensively in type and pathway involvement, their downstream immune response and cell death molecular events are typically conserved [28]. Consistent with this, KEGG enrichment of DEGs in this study revealed associations with plant–pathogen interactions, the MAPK signaling pathway, and ubiquitin-dependent protein binding (Figure 3A). Subsequent qRT-PCR analysis confirmed upregulated expression of defense-related genes, including *PR1* and *PWIR2*, after spot formation in the HSP, with *PR10* showing significant expression in the DS stage (Figure 3C). These findings reveal that spot formation in HSPs activated defense-related genes expression.

Notable differences in DEG numbers between HNPs and HSPs were observed across three stages, with down-regulated DEGs consistently outnumbering up-regulated ones during the SS and DS stages (Figure S3A). A Venn diagram analysis identified 123 overlapping DEGs on chromosome 3D between the SS and DS stages (Figure S3B). Volcano plots highlighted a predominance of down-regulated DEGs on chromosome 3D across all stages (Figures S3C–E).

### 2.5. Mapping of TaSpl1 to a 0.91 Mb Region on Chromosome 3DS

**Figure 4:**
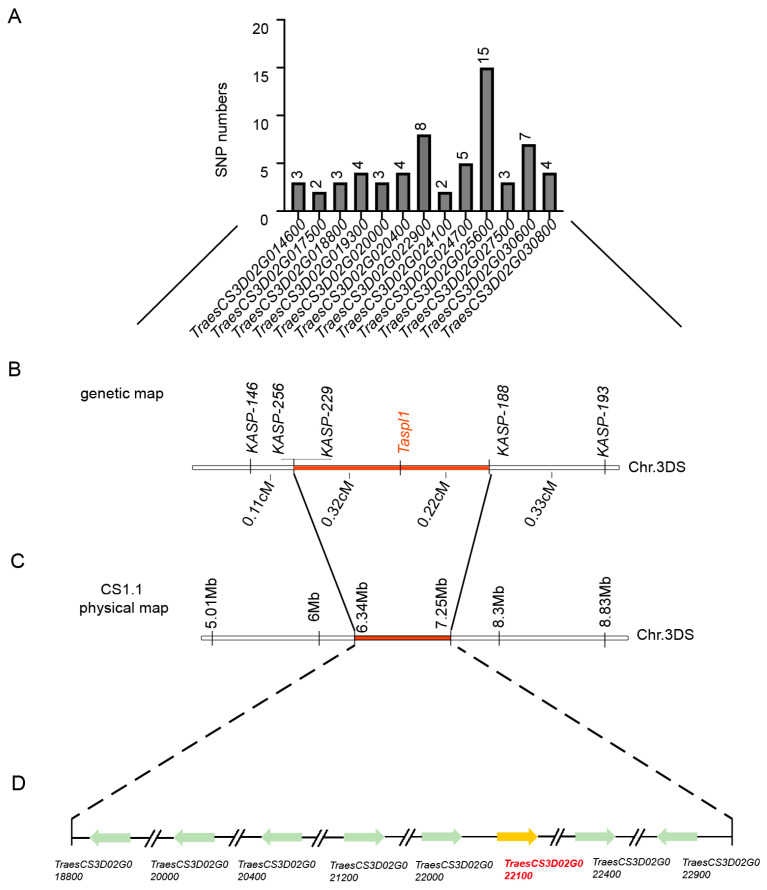
Fine mapping of spot gene *TaSpl1*. (**A**) Statistics of SNP information in the initial mapping interval according to BSR-Seq results. (**B**) Fine mapping of *TaSpl1* with two segregating populations including 199 plants and 918 plants by five KASP markers. (**C**) The location of the positioning interval in 3DS chromosome (6.34–7.25 Mb; IWGSC RefSeqv 1.1) on the physical map. (**D**) Eight DEGs between HNP and HSP in the fine mapping interval.

Previous studies showed SNP enrichment analysis using Euclidean distance from BSR-Seq identified a significant region with a maximum Euclidean distance of 2.0 at the distal end of chromosome 3DS [29]. Between markers SNP146 and SNP308, SNP distribution within interval genes was analyzed (Figure 4A, Table S3). To refine mapping, 5 KASP markers (Table S1) spanning the 5.01 to 11.58 Mb region were developed using the Chinese Spring reference genome v1.1. *TaSpl1* was ultimately mapped between *KASP188* and *KASP229*, with a genetic interval of 0.54 cM (Figure 4B). This candidate region, encompassing 0.91 Mb on chromosome 3DS (6.34–7.25 Mb; IWGSC RefSeq v1.1), contained 42 annotated genes, 14 of which were differentially expressed between HNPs and HSPs based on previous BSR-Seq data (Figure 4C). PCR amplification revealed base differences in eight genes, including *TraesCS3D02G018800*, *TraesCS3D02G020000*, *TraesCS3D02G020400*, *TraesCS3D02G021200*, *TraesCS3D02G022000*, *TraesCS3D02G022100*, *TraesCS3D02G022400*, and *TraesCS3D02G022900* (Figure 4D, Table S4).

### 2.6. Expression Analysis of DEGs in the Candidate Region by qRT-PCR

**Figure 5:**
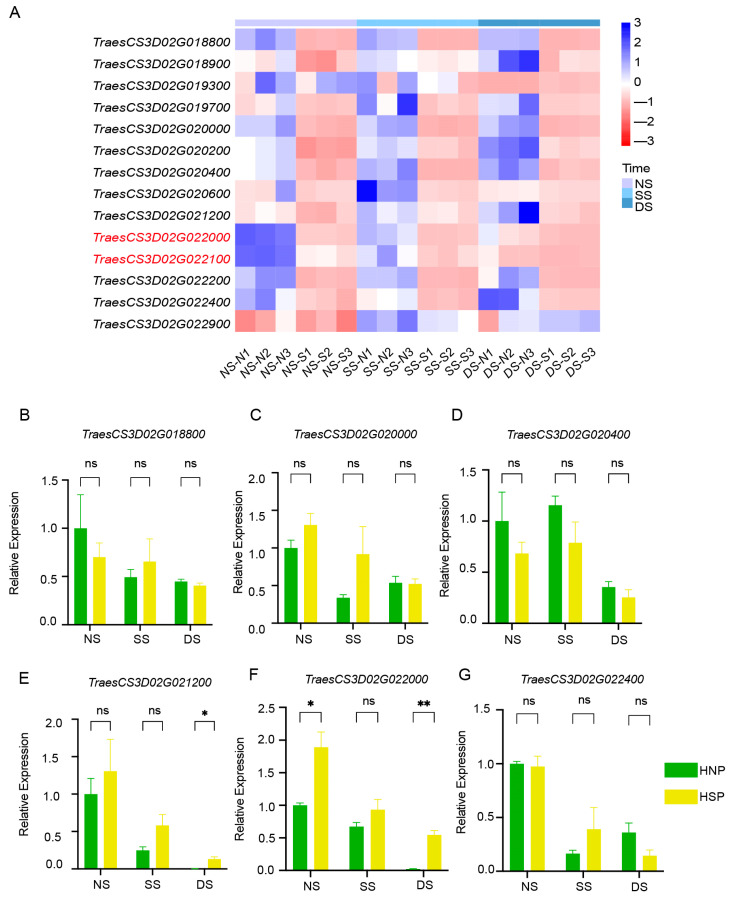
Analysis of DEGs in regions. (**A**) The heatmap of 14 DEGs between HNP and HSP in the fine mapping region of 0.54 cM in 3DS. (**B**–**G**) Relative expression of gene *TraesCS3D02G018800*, *TraesCS3D02G020000, TraesCS3D02G020400*, *TraesCS3D02G021200, TraesCS3D02G022000,* and *TraesCS3D02G022400* between HNP and HSP. ns, *p* > 0.05; *, *p* < 0.05; **, *p* < 0.01.

Heatmap analysis based on FPKM values indicated that *TraesCS3D02G022000* and *TraesCS3D02G022100* exhibited consistently downregulated expression trends (Figure 5A). qRT-PCR verification using primers (Table S1) for eight DEGs showed that most genes, including *TraesCS3D02G018800*, *TraesCS3D02G020000*, *TraesCS3D02G020400*, and *TraesCS3D02G022400*, exhibited no significant differential expression between HNPs and HSPs (Figure 5B–D,G). Gene *TraesCS3D02G021200* and *TraesCS3D02G022000* had significant differences between HNPs and HSPs. However, the expression of two genes in HSPs were higher than that in HNPs at three stages, which were inconsistent with the sequencing results of RNA-Seq (Figure 5A).

### 2.7. TraesCS3D02G022100 Is Associated with a Stay-Green Phenotype

**Figure 6:**
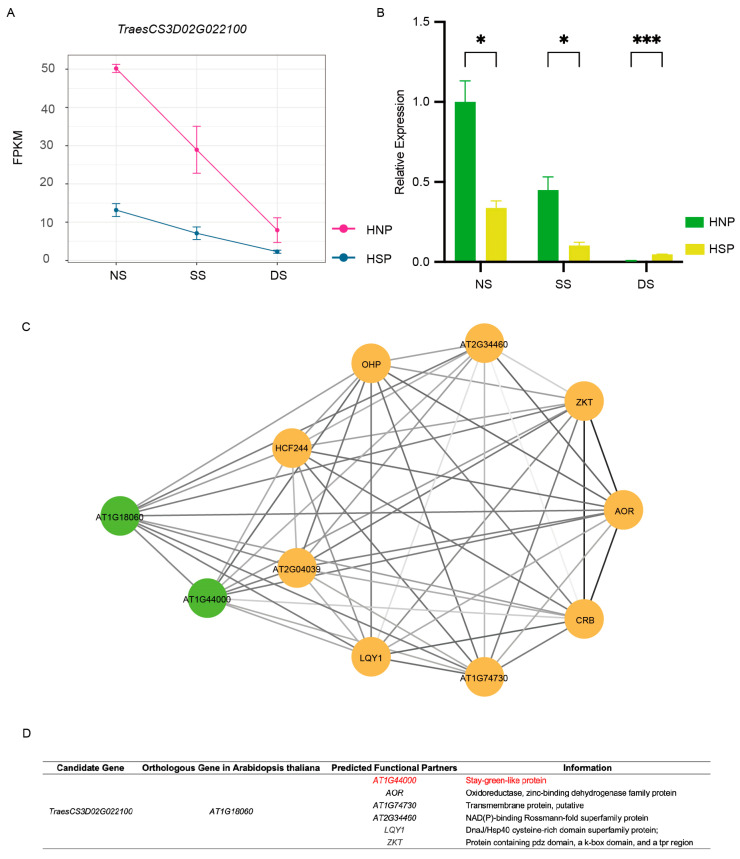
Gene analysis of *TraesCS3D02G022100*. (**A**) The FPKMs of gene *TraesCS3D02G022100* during spot formation period. (**B**) qRT-PCR of *TraesCS3D02G022100*. *, *p* < 0.05; ***, *p* < 0.001. (**C**) The network of homologous gene *AT1G18060* in *Arabidopsis* of *TraesCS3D02G022100*. Different circles represent different predicted functional partners. The color of the line segment is related to the co-expressed value. (**D**) The information of predicted functional partners information of *AT1G18060*.

*TraesCS3D02G022100* displayed a consistent downregulation trend across all three growth periods, which agreed with the FPKM results (Figure 6A,B). Conversely, *TraesCS3D02G022900* exhibited discrepancies between qRT-PCR and RNA-Seq data (Figure S4A,B). Associated fibrillin conserved domain-containing protein based on the UniProt Knowledgebase,; no functional studies on this gene have been reported thus far. Using the WheatOmics 1.0 HomologFinder tool, *AT1G18060* from *Arabidopsis* and *LOC_Os01g03040* from rice were identified as homologous genes of *TraesCS3D02G022100*. Notably, *AT1G18060* is chloroplast-localized and associated with a stay-green-like protein *AT1G44000* (Figure 6C,D). These findings identified *TraesCS3D02G022100* as a candidate gene for the spot phenotype.

**Figure 7:**
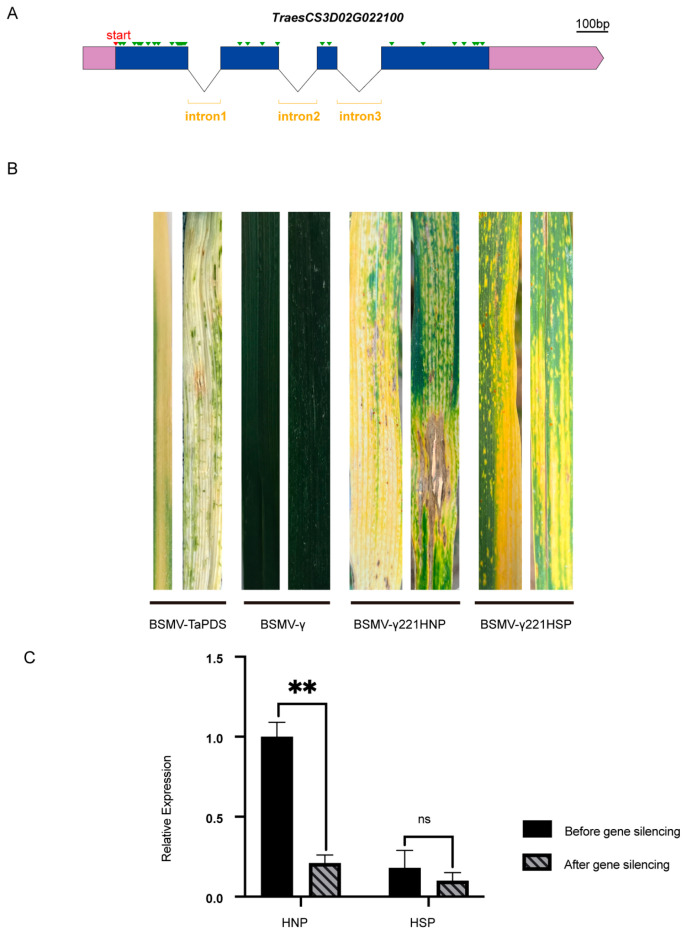
Gene structure and virus-induced gene silencing of candidate gene. (**A**) The gene structure of *TraesCS3D02G022100*. The green triangles represent single base difference between HNP and HSP. Scale bar, 100 bases. (**B**) The leaf color changes of BSMV-γ221HNP and BSMV -γ221HSP after 15 days of *TraesCS3D02G022100* silencing. BSMV-γ0 and BSMV-TaPDS were controls. (**C**) Comparison of gene silencing efficiency. ns, *p* > 0.05; **, *p* < 0.01.

After gene cloning and structural analysis, *TraesCS3D02G022100* was found to have a single transcript with 32 single base differences (Figure 7A and Figure S5A) and 12 amino acid variations (Figure S5B) between HNPs and HSPs. To investigate whether this gene was associated with the stay-green phenotype, its expression was knocked down in wheat using virus-induced gene silencing (VIGS) with BSMV. Constructs named BSMV-γ221HNP and BSMV-γ221HSP were successfully developed. Ten days after inoculation with BSMV-TaPDS, HNP leaves exhibited a silenced phenotype, confirming the efficacy of the BSMV system (Figure 7B). By day 20, HSP leaves displayed prominent spots, while HNP leaves inoculated with BSMV-γ221HNP exhibited discoloration and necrosis resembling a hypersensitive response. In HSPs, the spots merged into yellow patches following inoculation with BSMV-γ221HSP, suggesting accelerated leaf senescence (Figure 7B).

qRT-PCR analysis revealed an 82% reduction in *TraesCS3D02G022100* expression in HNPs and a 52.4% reduction in HSPs (Figure 7C). These findings indicate that silencing *TraesCS3D02G022100* accelerated leaf chlorosis, particularly in HNPs, implicating this gene associates with the stay-green phenotype.

## 3. Discussion

The identification and characterization of lesion mimic (LM) genes in crops have been challenging due to differences in chromosome composition, genome size, and the quality of reference genome assemblies across species. In wheat, LM-associated genes have been mapped to several chromosomes, including 1BL, 3BS, 4BL, 3BL, 2DS, 5DS, 3DS, 2AL, and 6BL, with examples such as *lm*, *lm1*, *lm2*, *lm3*, *lm4*, *Lmpa1* [30], *TaSpl1*, *lm5*, and *lm6*. Despite these discoveries, only a limited number of LM genes have been successfully cloned [19].

Abiotic factors, particularly light and temperature, are frequently considered critical in spot formation. Lesion mimic phenotypes in many reported cases are light-dependent, as observed in *lm2* [15], *lm3* [16], *lm5* [18], and *lm6* [19] in wheat, as well as *spl24* [31] and *spl40* [25] in rice. In this study, we observed yellow spots on the leaves of HSP plants (Figure 1B). Notably, although upper leaves receive more light due to their advantageous positioning and typically have higher photosynthetic efficiency, yellow spots consistently first appeared on the lower leaves. Shading experiments revealed that covered areas exhibited fewer spots (Figure 1C), suggesting that light may not be essential for spot formation. Our findings indicate that spot development in HSPs began at the booting stage regardless of whether plants were grown in the field or greenhouse. We hypothesize that the developmental stage of leaf cells plays a critical role in their sensitivity to light and the subsequent formation of spots. Once initiated, these spots persist and spread until plant senescence. This suggests that the booting stage marks a crucial window for cellular processes contributing to spot formation.

Programmed cell death (PCD) plays a crucial role in plant development, responses to abiotic stress, and immunity [32]. LMMs exhibit spontaneous PCD in the absence of pathogen infections [33]. Numerous studies have demonstrated a strong association between lesion mimic mutants and crop disease resistance. In rice, many lesion mimic mutants display enhanced pathogen resistance, often due to gene dysfunction that triggers immune stress responses, thereby bolstering defense mechanisms [23]. In this study, cell death and H_2_O_2_ accumulation in the spot cells of HSPs were confirmed through DAB and Trypan blue staining (Figure 1D,E), verifying that the spot phenotype is a type of lesion mimic mutation. ROS likely play a role in upregulating defense-related genes, disrupting bacterial membranes and preventing pathogen infection [18]. The spot formation in HSPs enhanced resistance to wheat stripe rust (mixed races CY32 and CY34) and powdery mildew (race *Bgt* E09) (Figure 2A,B). Importantly, agronomic trait analyses revealed that these spots had no significant impact on wheat yield (Figure 2G–I), suggesting that spot formation may activate a specific resistance mechanism without compromising productivity. Thus, integrating broad-spectrum disease resistance from lesion mimic mutants is a promising strategy for wheat breeding.

Gene expression analysis and functional enrichment are essential for understanding molecular responses. Previous studies have shown that DEGs are enriched in photosynthesis-related pathways through GO and KEGG analyses. For instance, the analyses have highlighted the strong positive selection of photosynthesis genes during the domestication of upland and lowland rice cultivars [34]. In this study, KEGG and GO enrichment analyses revealed that DEGs between HNPs and HSPs were significantly associated with photosynthesis, photosynthesis-antenna proteins, carbon fixation in photosynthetic organisms, and responses to light stimuli (Figure 3A,B). While previous studies on wheat lesion mimic mutants have focused on tissue-specific expression, this study leveraged RNA-Seq to analyze DEGs across three developmental stages, representing an important advancement in investigating lesion mimic mutants.

Stay-green-related genes have been extensively studied in *Arabidopsis*, cucumber [35], and rice [36]. In *Arabidopsis thaliana*, SGR homologs, including *SGR1*, *SGR2*, and *SGR-LIKE* (*SGRL*), have been identified [37]. Overexpression of *SGRL* causes early leaf yellowing, whereas *sgrl-1* mutants exhibit persistent green leaves under abiotic stress [38]. Based on functional predictions (Figure 6C,D), we hypothesize that *TaSpl1* acted as a stay-green gene that was downregulated from the booting stage until complete leaf yellowing in the HSP. Gene silencing experiments supported this hypothesis: HNP leaves inoculated with BSMV-γ221HNP exhibited severe discoloration and necrosis, while HSP leaves showed a milder phenotype (Figure 7B). qRT-PCR revealed higher expression levels and a more pronounced silencing effect for *TraesCS3D02G022100* in HNPs compared to HSPs, further corroborating its role in the stay-green phenotype.

## 4. Materials and Methods

### 4.1. Plant Materials

The plant materials used in this study included the wheat cultivar Xinong 509 (XN509) and line N07216, both with normal leaf phenotypes. The F_2_ segregating populations exhibited yellow spots, and the spot-related gene *TaSpl1* was identified as a dominant regulatory gene suppressed by two other dominant genes [29]. Through repeated selfing of F_3_ populations, we obtained homozygous normal plants (HNPs) and homozygous spotted plants (HSPs) by the F_8_ generation (Figure S1). HNPs and HSPs shared highly similar genetic backgrounds. Near-isogenic lines (NILs) included two segregating populations comprising 199 (F_7:8_) and 918 (F_8:9_) plants for fine mapping. All plant materials were cultivated with 20 seeds per row in 2-m rows at Northwest A&F University (121°12′46″ E, 41°2′53″ N).

### 4.2. Spot Phenotype and Light-Dependent Identification

Leaf phenotype variations in HNPs and HSPs were recorded and photographed from the seedling stage until maturity. Segregating populations were also evaluated for phenotypic variation during fine mapping. Given the well-established influence of light on plants [39], experiments were conducted to determine whether spots were light-dependent. Flag leaves from HNP and HSP plants were covered with 2.0 cm × 2.0 cm aluminum foil before spot appearance under controlled conditions (16/8 day/night, 5000 lux; 25 °C, 50% relative humidity). Foil covers were removed once spots fully developed to observe phenotypic changes.

### 4.3. Histochemical Characterization

Trypan blue staining was used to assess cell injury or death, as it penetrates disrupted cell membranes and binds to degraded DNA. Leaves from HNP and HSP plants were collected at the booting stage and immersed in boiling trypan blue staining solution for 20 min. Samples were decolorized with 2.5 mg/mL chloral hydrate (250 g in 100 mL water) at room temperature for 3–4 days before photographing [40].

Hydrogen peroxide (H_2_O_2_) was detected using 3,3′-diaminobenzidine (DAB) staining [3]. Leaves were immersed in a DAB solution and incubated for 2–6 h at room temperature in the dark. Chlorophyll was extracted by transferring the leaves to 95% ethanol at 40 °C until all chlorophyll was removed. Leaf samples were stored in fresh ethanol at 25 °C before photographing.

### 4.4. Evaluation of Disease Resistance

The wheat parents XN509, N07216, HNP, and HSP lines (40 plants each) were cultivated in a field at Northwest A&F University. Mixed races of *Puccinia striiformis* f. sp. *tritici* races CY32, and CY34 [41] were inoculated on 20 plants of each variety at the filling stage. The remaining plants were exposed to *Blumeria graminis* f. sp. *tritici* race E09 at 70% relative humidity [42] for powdery mildew assessment at Yangling. Inoculations were conducted as previously described [43]. Humidity was maintained until spore formation, coinciding with the appearance of spots. The disease resistance of materials was determined by statistical resistance grades. The experiment was conducted with three replicates in a randomized design.

### 4.5. Agronomic Performance Evaluation

Agronomic performance evaluations were conducted in the 2022–2023 growing season at Yangling. A total of 50 HNP and HSP plants were randomly selected for assessment of plant height (PH), spike length (SL), spike number per plant (SNP), thousand-grain weight (TGW), grain number per spike (GNS), grain weight per plant (GWP), grain length (GL), and grain width (GW) upon wheat maturation. Statistical analysis was performed using SPSS software R25 0.0.2 [17] to identify significant differences.

### 4.6. Submicroscopic Structure Observation of Chloroplast

To investigate the effects of the chloroplast structure on photosynthesis, flag leaves from HNP and HSP plants at the flowering stage were collected for submicroscopic structure analysis. Leaf segments (2 mm × 5 mm) were cut along the veins, and yellow-spotted regions of HSP leaves were marked. Samples were fixed in 4% (*w*/*v*) glutaraldehyde [44], under vacuum for more than 6 h. They were subsequently washed with PBS, dehydrated in a gradient series, infiltrated, embedded, and polymerized in an oven at 58 °C. Polymerized samples were trimmed and cut into 50–70 nm sections. After double staining with 2% uranyl acetate and lead citrate, chloroplast morphology and localization were observed using transmission electron microscopy.

### 4.7. Comparison of Photosynthetic Pigment Content and Determination of Photosynthesis

Chlorophyll is a key pigment essential for photosynthesis in green plants, and variations in its content directly influence leaf color and photosynthetic efficiency [45]. Chlorophyll a and b levels were measured using flag leaves from HNPs and HSPs 2 weeks after anthesis. Each 0.3 g leaf sample was finely cut and soaked in 20 mL of 80% acetone at 4 °C for 48 h in darkness until fully decolorized. Absorbance at 663 nm and 645 nm was measured using a spectrophotometer (UV-1700, Macylab Instrument Inc, Shanghai, China), with 80% acetone serving as the blank. Chlorophyll concentrations were calculated using standard equations [46]. Three biological replicates were assessed for each sample.

To evaluate photosynthetic efficiency, measurements of photosynthesis were conducted between 9:00 and 11:00 a.m. on a clear day. A total of 12 plants from each genotype (XN509, N07216, HNP, and HSP) were selected. Flag leaves were detached for measurement of the photosynthetic rate (Pn), stomatal conductance (Gs), intercellular CO_2_ concentration (Ci), transpiration rate (Tr), and water use efficiency (WUE) using a Li-6400 photosynthesis system (LI-COR Corporate) with a standard leaf chamber [47]. SPSS and GraphPad Prism 9.4.1 were employed for statistical analyses [17].

### 4.8. Differential Gene Expression Analysis by RNA-Seq

An RNA-Seq was performed to investigate gene expression differences between HNPs and HSPs across three developmental stages: no spots (NSs) at the booting stage, some spots appearance (SSs) stage, and distributed spots (DSs) stage. Samples were prepared in triplicate and sent to the Biomarker Technology Co., Ltd. (Beijing, China) for RNA-Seq, including RNA quality assessment, library construction, and sequencing. Gene expression levels were calculated based on FPKM values following established methods [48]. Differentially expressed genes (DEGs) were identified, and their expression patterns were analyzed statistically to elucidate regulatory mechanisms underlying the leaf spot phenotype.

### 4.9. Fine Mapping of TaSpl1

Previous research localized the *TaSpl1* spot gene near the terminal region of chromosome 3D between SNP markers *SNP145* and *SNP256* using BSR-Seq data [29]. For fine mapping, two segregating populations comprising 199 and 918 plants were developed from crosses between XN509 and N07216. KASP markers were designed using PolyMarker (https://www.polymarker.info/ (accessed on 15 November 2023)) [49] to refine the target region. Genomic DNA was extracted from XN509, N07216, HNP, HSP, and the segregating populations using the CTAB method. Genotypic and phenotypic data were analyzed using Klustering Caller software 4.1.2 [50] and marker distances from *TaSpl1* were calculated using IciMapping 4.2 to construct the genetic map.

### 4.10. Quantitative Reverse-Transcriptase Polymerase Chain Reaction (qRT-PCR)

Specific primers for qRT-PCR were designed using SnapGene software 6.0.2. Total RNA was extracted using TRIzol reagent (Thermo Scientific, Waltham, MA, USA) from samples flash-frozen in liquid nitrogen. First-strand cDNA synthesis was carried out using PrimeScript™ RT reagent Kit (Takara Biotechnology, Dalian, China). The 10 µL qRT-PCR reaction mixture included 2 µL cDNA template (250 ng), 5 µL TB Green Premix Ex Taq (Tli RNaseH Plus), 0.5 µL forward primer (10 µmol/µL), 0.5 µL reverse primer (10 µmol/µL), and 2 µL DNase/RNase-free water. Amplification was performed on a Roche LightCycler^®^480 II Real-Time System with the following thermal profile: initial denaturation at 95 °C for 10 min, followed by 40 cycles at 95 °C for 10 s, 60 °C for 30 s, and 72 °C for 30 s, and a final dissociation step at 95 °C for 15 s, 60 °C for 30 s, and 95 °C for 15 s. Tatubulin was used as the reference gene, and relative gene expression was calculated using the 2^−ΔΔCT^ method [51].

Considering previous findings that certain lesion mimic mutants exhibit adult-plant resistance to powdery mildew [16]. The expression of four defense-related genes (*PR1* [52], *PR4*, *PR10*, and *PWIR2* [53]) was analyzed in HNPs and HSPs using qRT-PCR. Experimental steps were identical to those described above. Primers used for qRT- PCR are listed in Table S1.

### 4.11. Gene Analysis and Silencing

The CDS sequences of candidate genes from HNPs and HSPs were obtained from the AuGCT DNA-SYN Biotechnology Co., Ltd. (Yangling, Xianyang, China). Protein information was retrieved from the UniProt knowledge base (https://www.uniprot.org (accessed on 23 January 2024)), while homologous gene information was sourced from WheatOmics (http://wheatomics.sdau.edu.cn (accessed on 25 January 2024)). Protein interaction predictions were conducted using STRING (https://cn.string-db.org/ (accessed on 25 January 2024)).

The barley stripe mosaic virus (BSMV) RNA-induced gene silencing (VIGS) system was employed to silence candidate genes. Fragments of the target genes from HNPs and HSPs were amplified, sequenced, and inserted into specific plasmids through homologous recombination using the ClonExpress II One Step Cloning Kit (Vazyme). The gene fragments were ligated with BSMV-γ after digestion with *BamH*I and *Spe*I. The empty γ0 vector and BSMV-TaPDS, targeting the wheat phytoene desaturase (PDS) gene, were used as controls. Gene silencing was induced through tobacco transcription [51] before the appearance of spots in HNPs and HSPs under field conditions. Leaf phenotypes were documented, and gene silencing efficiency was evaluated by qRT-PCR.

## 5. Conclusions

In conclusion, this study provided a deeper insight into the genetic basis of the lesion mimic mutation. RNA-Seq analysis across three developmental stages revealed that DEGs were enriched in photosynthesis-related pathways between HNPs and HSPs. The *TaSpl1* gene was fine-mapped to a 0.91 Mb interval on chromosome 3DS using two segregating populations derived from a cross between XN509 and N07216. Candidate gene sequence analysis and gene silencing experiments demonstrated that *TraesCS3D02G022100* was associated with the stay-green phenotype. These findings offer valuable insights into spot mutants resembling lesion mimic mutants and provide a novel genetic resource for enhancing disease resistance in wheat.

## Figures and Tables

**Table 1:** Infection types of wheat disease resistance.

Races	XN509	N07216	HNP	HSP
*Pst* CYR 32	3	3	2	0;
*Pst* CYR 34	3	3	2	0;
*Bgt* E09	3	2	3	0

Grades ”2” and “3” represent susceptible; Grade ”0” represents resistant.

**Table 2:** Comparison of photosynthesis-related indexes of XN509, N07216, HNP, and HSP.

Variety	Pn (μmol·m^−2^·s^−1^)	Gs (mmol·m^−2^·s^−1^)	Ci (μmol·m^−1^)	Tr (mmol·m^−2^·s^−1^)	WUE (μmol·mmol^−1^)
XN509	16.81 ± 2.11 ^B^	0.49 ± 0.06 ^A^	322.92 ± 8.63 ^A^	7.85 ± 0.59 ^A^	2.14 ± 0.21 ^C^
N07216	15.76 ± 1.72 ^B^	0.35 ± 0.08 ^B^	305.25 ± 4.72 ^B^	7.71 ± 1.12 ^A^	2.06 ± 0.25 ^C^
HNP	19.33 ± 1.97 ^A^	0.53 ± 0.04 ^A^	324.25 ± 7.81 ^A^	6.44 ± 0.37 ^B^	3 ± 0.31 ^A^
HSP	15.46 ± 1.05 ^B^	0.38 ± 0.04 ^B^	317.75 ± 6.08 ^A^	6.02 ± 0.35 ^B^	2.57 ± 0.19 ^B^
Average	16.84	0.4375	317.48	7.005	2.45
*F*	11.86 **	26.85 **	9.58 **	22.92 **	39.06 **
CV%	10.44%	19.70%	2.71%	13.03%	17.77%

Pn represents photosynthetic rate, Gs represents conductance, Ci represents intercellular CO_2_ concentration, Tr represents transpiration rate, WUE represents Water Use Efficiency. Different letters represent the significance difference, *p* < 0.05. ** represents *p* < 0.01.

## Data Availability

The raw sequence data reported in this paper have been deposited in the Genome Sequence Archive in the National Genomics Data Center, China National Center for Bioinformation/Beijing Institute of Genomics, Chinese Academy of Sciences (GSA: CRA021671) that are publicly accessible at https://ngdc.cncb.ac.cn/gsa.

## Supplementary Materials

The following supporting information can be downloaded at: https://www.mdpi.com/article/10.3390/ijms26094002/s1.
