# Supplementary material for: Characterization and Fine Mapping of the Stay-Green-Related Spot Leaf Gene TaSpl1 with Enhanced Stripe Rust and Powdery Mildew Resistance in Wheat
Source: Int J Mol Sci. 2025 Apr 23;26(9):4002. doi: 10.3390/ijms26094002 (PMC12071801; doi:10.3390/ijms26094002)
Supplement: Supplementary file 1 [file ijms-26-04002-s001.zip › Supplementary Figures.pdf]

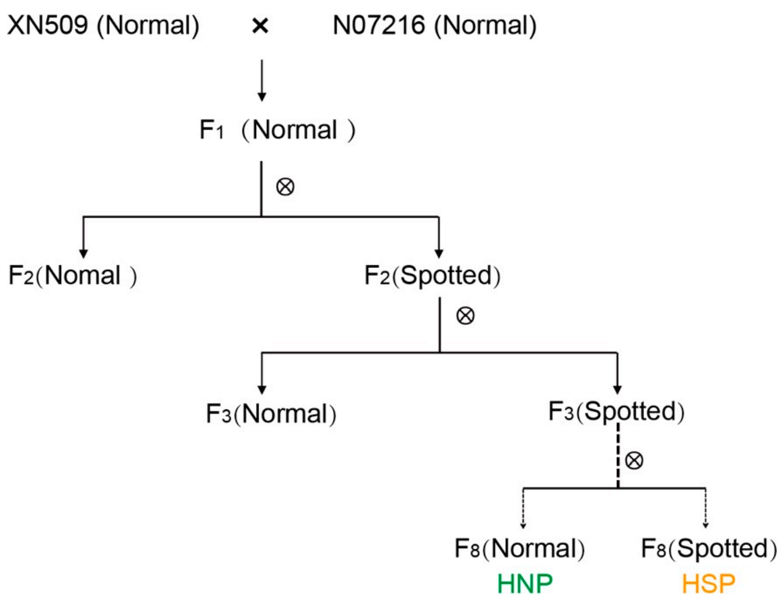

Figure S1. The sources of plant materials HNP and HSP. The parents XN509 and N07216 both showed nomal green leaves. F_2 population appeared segregation of leaf color with yellow spots. Due to the existence of inhibitory genes, HNP and HSP were obtained by repeated selfing by selecting appropriate segregation lines with appropriate segregation ratios.

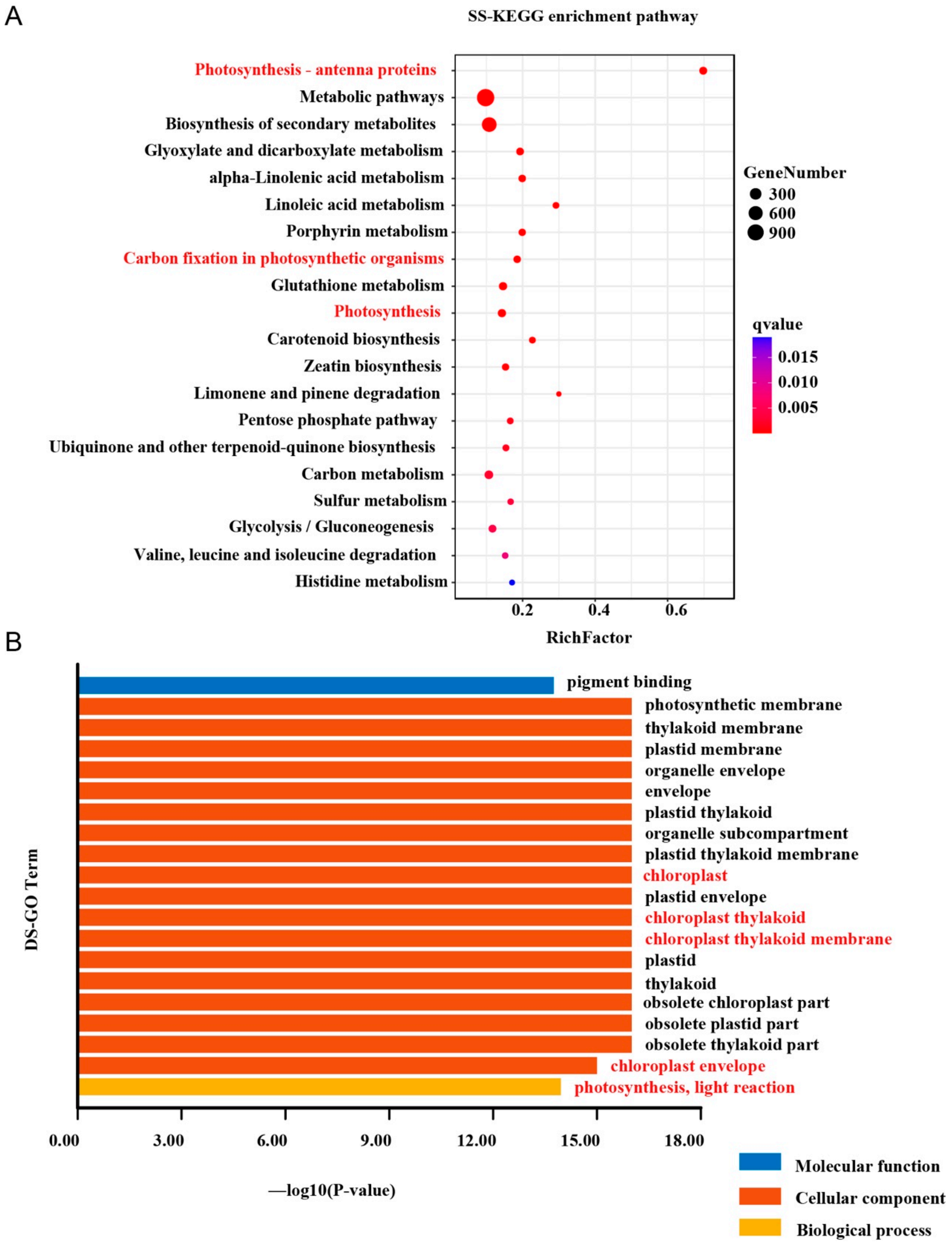

Figure S2. Enrichment analysis of DEGs in DS stage. (A) KEGG enrichment of DEGs between HNP and HSP in DS stage. (B) GO enrichment of down-regulated DEGs between HNP and HSP in DS stage.

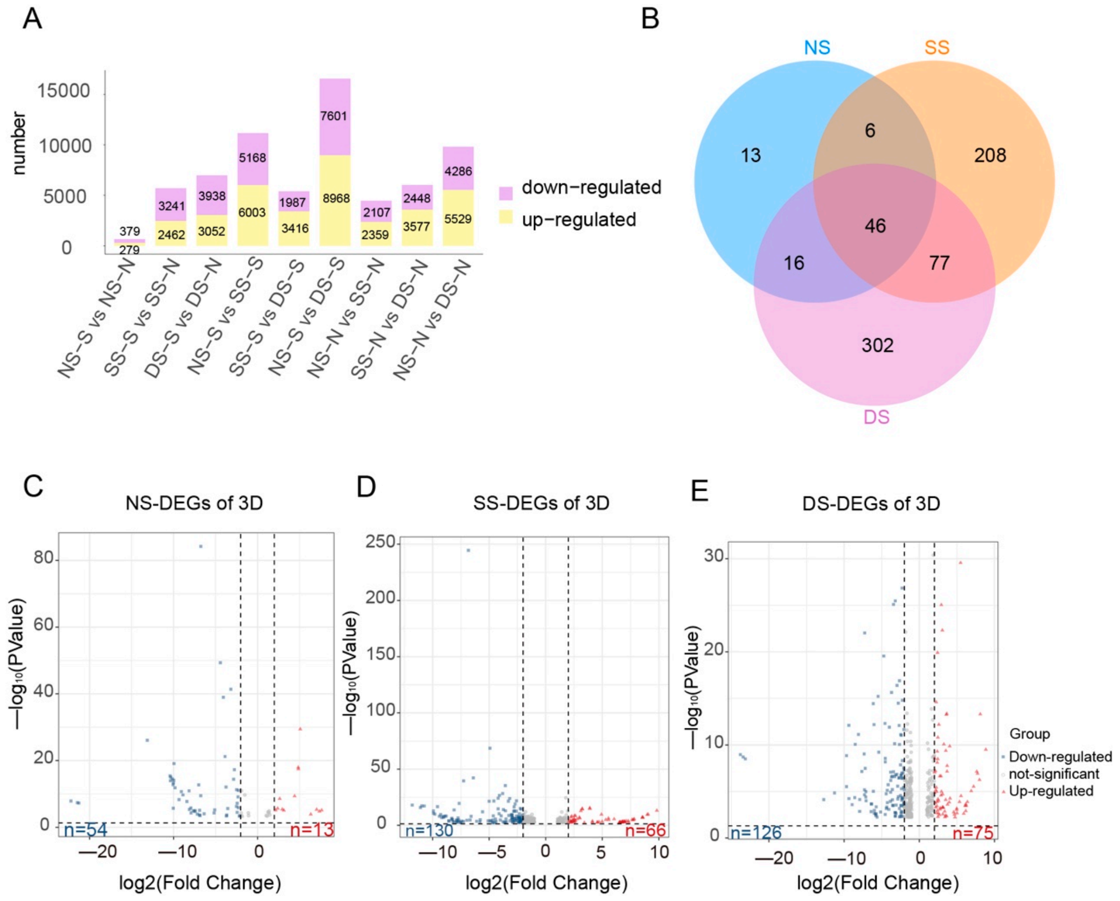

Figure S3. The comparation of DEGs between HNP and HSP. (A) Statistics of the numbers of DEGs. N represent HNP, S represent HSP, (B) The count of DEGs in 3D chromosome common to HNP and HSP in NS, SS and DS stages. (C) Volcano plot of DEGs in 3D chromosome for HNP and HSP in NS stage. (D) Volcano plot of DEGs in 3D chromosome for HNP and HSP in SS stage. (E) Volcano plot of DEGs in 3D chromosome for HNP and HSP in DS stage.

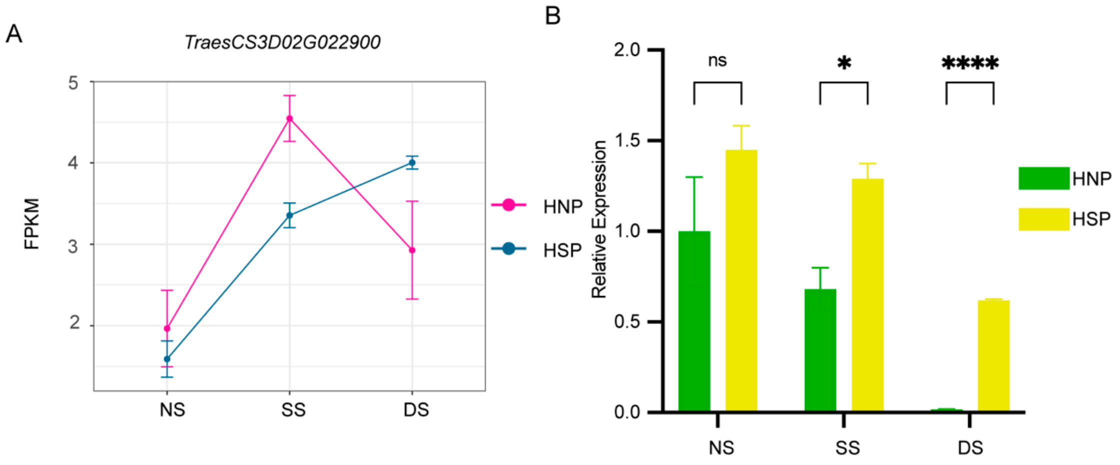

Figure S4 Gene analysis of *TraesCS3D02G022900*. (A)The FPKMs of gene *TraesCS3D02G022900* by RNA-Seq during spot formation period. (B)qRT-PCR of gene *TraesCS3D02G022900*.

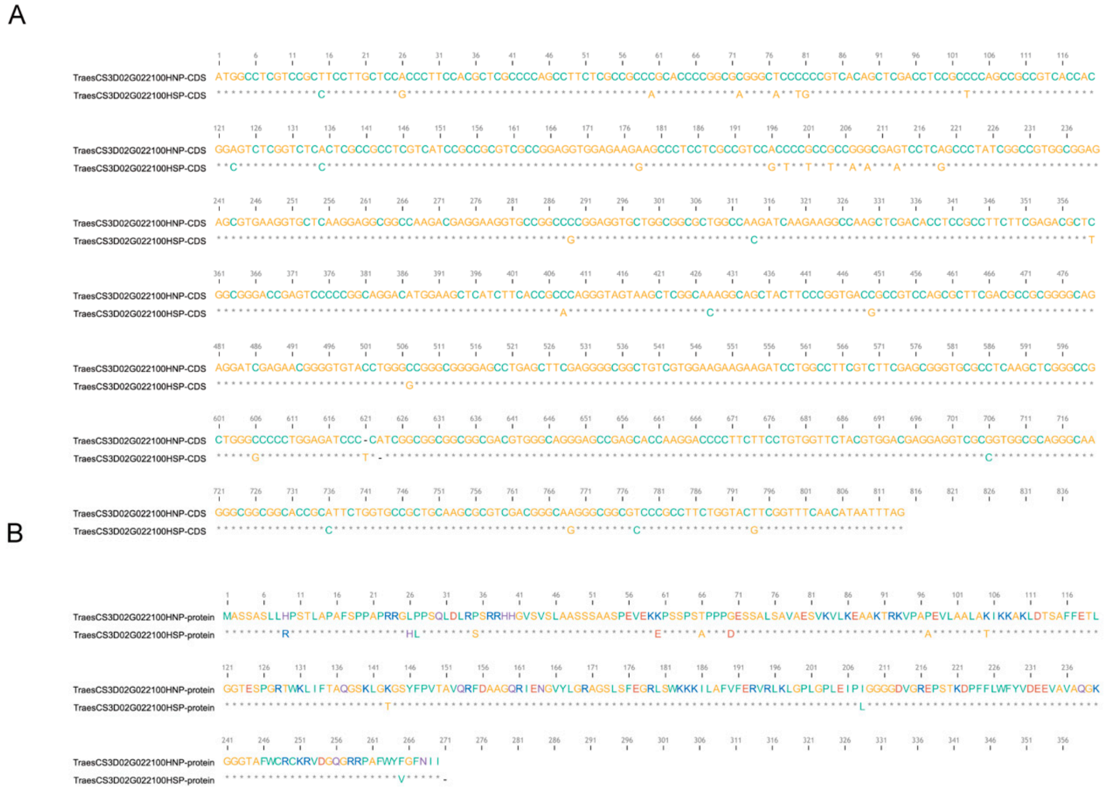

Figure S5. Gene analysis of candidate genes. (A) CDS sequence analysis of candidate gene *TraesCS3D02G022100* between HNP and HSP. * represents conserved base. (B) Amino acid sequence analysis of *TraesCS3D02G022100* between HNP and HSP. * represents conserved amino acid.
