# Supplementary material for: Characterization and Fine Mapping of the Stay-Green-Related Spot Leaf Gene TaSpl1 with Enhanced Stripe Rust and Powdery Mildew Resistance in Wheat
Source: Int J Mol Sci. 2025 Apr 23;26(9):4002. doi: 10.3390/ijms26094002 (PMC12071801; doi:10.3390/ijms26094002)
Supplement: Supplementary file 1 [file ijms-26-04002-s001.zip › Supplementary Tables.pdf]

Table S1 List of primers used in this study

Primer type	Primer name		Primer sequence
**List of primers used for fine mapping of *Taspl1***			
		Forward-1	5'-GAAGGTGACCAAGTTCATGCTACAATGGTATGTACATGCCTGT-3'
	KASP145	Forward-2	5'-GAAGGTCGGAGTCAACGGATTACAATGGTATGTACATGCCTGC-3'
		Reverse	5'-GTCATGGCCAAGTACCCACA-3'
		Forward-1	5'-GAAGGTGACCAAGTTCATGCTACTGGTCATATCGTCCCACAAA-3'
	KASP188	Forward-2	5'-GAAGGTCGGAGTCAACGGATTACTGGTCATATCGTCCCACAAG-3'
		Reverse	5'-AGGAGCGTATGGTACCAGCT-3'
		Forward-1	5'-GAAGGTGACCAAGTTCATGCTAAAGACCTCGTTAAGCTGATCAGGA-3'
KASP primers	*KASP193*	Forward-2	5'-GAAGGTCGGAGTCAACGGATTAAAGACCTCGTTAAGCTGATCAGGG-3'
		Reverse	5'-GTGCCAGGTACCTGCTCCTTG-3'
		Forward-1	5'-GAAGGTGACCAAGTTCATGCTCCCCCTTAATGTAGACGTTCATAC-3'
	KASP229	Forward-2	5'-GAAGGTCGGAGTCAACGGATTCCCCCTTAATGTAGACGTTCATAA-3'
		Reverse	5'-TGCGTGAGATGAGGGTTCTC-3'
		Forward-1	5'-GAAGGTGACCAAGTTCATGCTGCTTGGTAGCATCTCAGAATTTTTG-3'
	KASP256	Forward-2	5'-GAAGGTCGGAGTCAACGGATTGCTTGGTAGCATCTCAGAATTTTTA-3'
		Reverse	5'-ACCGATAGAGACTCAGGTGAC-3'
**List of primers used for qRT-PCR of DEGs**			
	qTraesCS3D02G018800	q188-F	5'-CAAGCACCAACTGGCTCTGT-3'
		q188-R	5'-CAGTTCGTTGTCCGCATTTT-3'
qRT-PCR primers	*qTraesCS3D02G020000*	q200-F	5'-GTCCGCATCCTTGCTCCAC-3'
		q200-R	5'-GGCACCTTCCTCGTCTTGG-3'
	qTraesCS3D02G020400	q204-F	5'-GGGATCTCTCGTTTGCGAGG-3'

		q204-R	5'-TCATATCACGGGTGGCACAA-3'
	qTraesCS3D02G021200	q212-F	5'-CTGAAATGGCAGCAGAAACT-3'
		q212-R	5'-CTCCTCCTCTTTGACCTGTG-3'
	qTraesCS3D02G022000	q220-F	5'-TCAAGGACGCGGCCAAGACG -3'
		q220-R	5'-ACCCTGGGCGGTGAAGATGAGC-3'
	qTraesCS3D02G022100	q221-F	5'-CACGGAGTCTCGGTCTCAC-3'
		q221-R	5'-TGTCGAGCTTGGCCTTCTTG-3'
	qTraesCS3D02G022400	q224-F	5'-TATTGGGGCGAACCTTTCCC-3'
		q224-R	5'-CTCTTCTTGCAGGCTTCCCA-3'
	qTraesCS3D02G022900	q229-F	5'-GGAATCCATTGAGCTTGGGC-3'
		q229-R	5'-TCGGATTCTATATCTGGCCTCCT-3'
	qTaTubulin	Tu-F	5'-CGTGCTGTCTTTGTAGATCTCG-3'
		Tu-R	5'-GACCAGTGCAGTTGTCTGAAAG-3'
**List of primers used for qRT-PCR**			
defense-related gene primers	*PR1*	qPR1-F	5'-CTGGAGCACGAAGCTGCAG-3'
		qPR1-R	5'-CGAGTGCTGGAGCTTGCAGT-3'
	PR4	qPR4-F	5'-CGAGGATCGTGGACCAGTG-3'
		qPR4-R	5'-GTCGACGAACTGGTAGTTGACG-3'
	PR10	PR10-F	5'-TTAAACCAGCACGAGAAACATCAG-3'
		PR10-R	5'-ATCCTCCCTCGATTATTCTCACG-3'
	PWIR2	PWIR2-F	5'-AGGTAATTTTTTTATTGCCCTGTACTG-3'
		PWIR2-R	5'-TTACAGCCGCCGTACTACATGT-3'
**List of primers used for CDS cloning**			
CDS cloning	*TraesCS3D02G018800*	188-F	5'-ATTCGTAACCTTCCCGATTTCTTGA-3'

	188-R	5'-AGATATGTGAACCGCACACAACAAC-3'
TraesCS3D02G018900	189-F	5'-CTGCTCTGCTCGGCTCGG-3'
	189-R	5'-CCGGAAAGTGCATGCAGCCT-3'
TraesCS3D02G019300	193-F	5'-GAAAATGAGCGTAATCGTAACCAAG-3'
	193-R	5'-GTCAGGTGAACTTGGCCAGCT-3'
TraesCS3D02G019700	197-F	5'-ATGGGCGAAGGGGCTGC-3'
	197-R	5'-TGTCTGTCACGGGGAGCAGTC-3'
TraesCS3D02G020000	200-F	5'-GCGGGCATGGCGGGGAT-3'
	200-R	5'-TCATGTATGCAGGAAAGCAACGGTG-3'
TraesCS3D02G020200	202-F	5'-ATGGCGACCTTCGCGAAG-3'
	202-R	5'-AGGCCAGGCAGCTTCAGC-3'
TraesCS3D02G020400	204-F	5'-CGCCAGATCCCTGTCCAATG-3'
	204-R	5'-CATTCTCTTCCGCGACCTATTTATG-3'
TraesCS3D02G020600	206-F	5'-GGACGAGCGGCGGAAATG-3'
	206-R	5'-CGAACGTCATGTTCATTGCCCT-3'
TraesCS3D02G021200	212-F	5'-ATGGATGCCAAGAAGTTCCT-3'
	212-R	5'-TTACACGAGTTGCTTCCCGT-3'
TraesCS3D02G022000	220-F	5'-AAGATGGCCTCGTCCGCA-3'
	220-R	5'-ACCTTGGTCGTCATGCCG-3'
TraesCS3D02G022100	221-F	5'-AAGAGGAGGAAGAAGATGGCC-3'
	221-R	5'-GGACGCTGGCTGATCTATCTAAATT-3'
TraesCS3D02G022200	222-F	5'-AGCTTCTCCGCGGGGATG-3'
	222-R	5'-CGAGAGTTGCGCAGAAAAAATTAC-3'
TraesCS3D02G022400	224-F	5'-AGCTTCTCCGCGGGGATG-3'

		224-R	5'-TCACGTCCTTGCCTTTTGTTG-3'
	TraesCS3D02G022900	229-F	5'-CGATGGAGACGGAGGAGC-3'
		229-R	5'-TGCAACCGTCAGCTGCCT-3'
	M13	M13-F	5'-TGTAAAACGACGGCCAGT-3'
		M13-R	5'-CAGGAAACAGCTATGACC-3'
**List of primers used for construction of BSMV vectors**			
	IF-TraesCS3D02G022100	IF221-F	5'-GGCAGAGGATTGAGAACGGG-3'
		IF221-R	5'-CTCGTCGACGTAGAACCACA-3'
PDS	*PDS-IF-TraesCS3D02G022100*	PDS-IF221-F	5'-CTTCTTCCGTTTCTAAGTAAACTAGTGGCAGAGGATTGAGAACGGG-3'
		PDS-IF221-R	5'-CTTGCTATTTCTAGCTCTAAGGATCCCTCGTCGACGTAGAACCACA-3'
	TYYW	TYYW-F	5'-GATCAACTGCCAATCGTGAGTAGG-3'
		TYYW-R	5'-GTGGACTTGCAAACACTCCCATC-3'

Table S2 summary of RNA-Seq reads results

Sample ID	Obtained Reads	OBtained Base(bp)	Q20(%)	Q30(%)	GC(%)
NP-HNP-R1	33,203,653	9,913,774,282	97.78	94.19	52.84
NP-HNP-R2	29,231,578	8,738,374,930	97.74	94.12	52.77
NP-HNP-R3	28,492,336	8,513,367,500	97.6	93.79	52.48
NP-HSP-R1	30,286,523	9,034,451,268	97.66	93.94	53.37
NP-HSP-R2	27,995,378	8,354,050,718	97.79	94.24	53.09
NP-HSP-R3	31,175,742	9,303,657,924	97.31	92.94	52.3
SP-HNP-R1	30,737,627	9,127,894,292	96.92	92.48	50.69
SP-HNP-R2	29,075,220	8,677,077,912	97.17	92.77	53.1
SP-HNP-R3	26,610,135	7,927,216,938	98.03	94.84	52.4
SP-HSP-R1	30,773,484	9,198,193,936	97.82	94.21	52.14
SP-HSP-R2	31,309,050	9,364,311,728	97.75	94.08	51.83
SP-HSP-R3	30,900,147	9,226,919,270	97.86	94.3	51.77
DP-HNP-R1	29,065,706	8,690,071,756	97.71	93.94	53.71
DP-HNP-R2	28,510,444	8,510,119,302	97.59	93.69	51.38
DP-HNP-R3	27,693,261	8,281,021,586	97.59	93.67	52.09
DP-HSP-R1	32,281,239	9,640,723,410	97.62	93.85	52.31
DP-HSP-R2	29,584,469	8,842,574,244	97.66	93.96	52.12
DP-HSP-R3	29,057,436	8,672,000,604	97.58	93.88	52.65

Table S3 SNP information in the initial mapping interval according to BSR-Seq results

Gene_id	Pos	Ref	Alt	HSP	Depth	AlleDp	HNP	Depth	AlleDp	Codon_change
TraesCS3D02G014600	5013682	G	A	A	31	0,31	N	.	.	Gca/Aca
TraesCS3D02G014600	5013938	A	G	G	13	0,13	N	.	.	aAc/aGc
TraesCS3D02G014600	5013962	A	T	T	6	0,6	N	.	.	aAa/aTa
TraesCS3D02G017500	6009303	G	A	R	1	0,1	G	13	13,0	gGg/gAg
TraesCS3D02G017500	6010815	T	C	Y	1	0,1	T	18	18,0	tgT/tgC
TraesCS3D02G018800	6343095	A	T	W	1	0,1	A	43	43,0	Tca/Aca
TraesCS3D02G018800	6343153	C	A	A	8	0,8	C	48	48,0	gcG/gcT
TraesCS3D02G018800	6343388	T	C	C	14	0,14	T	44	44,0	Acg/Gcg
TraesCS3D02G019300	6455100	A	C	M	1	0,1	A	55	55,0	gaT/gaG
TraesCS3D02G019300	6455111	T	C	C	6	0,6	T	58	58,0	Agc/Ggc
TraesCS3D02G019300	6455789	C	T	T	7	0,7	C	59	55,4	Gcc/Acc
TraesCS3D02G019300	6455840	T	C	C	19	0,19	Y	56	35,21	Aac/Gac
TraesCS3D02G020000	6730500	A	G	R	1	0,1	A	23	23,0	tTg/tCg
TraesCS3D02G020000	6732993	A	G	G	2	0,2	A	21	21,0	gtT/gtC
TraesCS3D02G020000	6732999	G	A	A	2	0,2	G	22	22,0	ccC/ccT
TraesCS3D02G020400	6862084	T	C	Y	1	0,1	T	7	7,0	Aat/Gat
TraesCS3D02G020400	6862998	G	C	C	3	0,3	G	6	6,0	aCc/aGc
TraesCS3D02G020400	6863095	T	C	C	2	0,2	T	5	5,0	Acc/Gcc
TraesCS3D02G020400	6863123	A	G	G	2	0,2	A	5	5,0	ggT/ggC
TraesCS3D02G022900	7257795	C	A	M	1	0,1	C	10	10,0	aGt/aTt

TraesCS3D02G022900	7258970	T	C	Y	1	0,1	T	16	16,0	tcA/tcG
TraesCS3D02G022900	7259555	G	A	A	11	0,11	G	22	22,0	gtC/gtT
TraesCS3D02G022900	7260053	G	A	A	16	0,16	G	14	14,0	atC/atT
TraesCS3D02G022900	7260633	G	A	A	8	0,8	G	17	17,0	tgC/tgT
TraesCS3D02G022900	7260651	A	T	T	5	0,5	A	12	12,0	gaT/gaA
TraesCS3D02G022900	7262895	A	G	G	2	0,2	A	5	5,0	Ttg/Ctg
TraesCS3D02G022900	7262929	G	A	A	3	0,3	G	6	6,0	gaC/gaT
TraesCS3D02G022900	7263025	T	C	C	4	0,4	T	7	7,0	ggA/ggG
TraesCS3D02G022900	7266423	G	C	C	3	0,3	G	7	7,0	Ctt/Gtt
TraesCS3D02G024100	7608489	T	G	G	2	0,2	T	5	5,0	gAa/gCa
TraesCS3D02G024100	7608500	T	C	C	2	0,2	T	5	5,0	atA/atG
TraesCS3D02G024700	8336557	A	G	G	3	0,3	A	70	70,0	Ttt/Ctt
TraesCS3D02G024700	8341130	C	G	G	6	0,6	C	72	72,0	gaG/gaC
TraesCS3D02G024700	8341196	G	C	C	14	0,14	G	65	65,0	agC/agG
TraesCS3D02G024700	8341277	A	G	G	16	0,16	A	49	49,0	taT/taC
TraesCS3D02G024700	8341341	G	A	A	14	0,14	G	36	36,0	gCt/gTt
TraesCS3D02G025600	8838648	G	C	S	3	1,2	G	77	77,0	gcC/gcG
TraesCS3D02G025600	8838667	G	A	R	5	1,4	G	72	72,0	gCg/gTg
TraesCS3D02G025600	8838861	C	T	Y	3	1,2	C	54	54,0	acG/acA
TraesCS3D02G025600	8839023	C	T	T	2	0,2	C	77	77,0	acG/acA
TraesCS3D02G025600	8839712	G	T	T	10	0,10	G	81	81,0	atC/atA
TraesCS3D02G025600	8839945	C	T	T	12	0,12	C	33	33,0	gcG/gcA
TraesCS3D02G025600	8840035	G	A	A	6	0,6	G	39	39,0	gtC/gtT

TraesCS3D02G025600	8840210	C	T	T	4	0,4	C	32	32,0	tcG/tcA
TraesCS3D02G025600	8840491	G	A	A	8	0,8	G	18	18,0	agC/agT
TraesCS3D02G025600	8840575	T	A	A	8	0,8	T	13	13,0	ctA/ctT
TraesCS3D02G025600	8840626	G	A	A	6	0,6	G	12	12,0	ggC/ggT
TraesCS3D02G025600	8841124	T	A	W	1	0,1	T	8	8,0	ggA/ggT
TraesCS3D02G025600	8841277	C	T	Y	1	0,1	C	12	12,0	ttG/ttA
TraesCS3D02G025600	8841376	C	G	G	2	0,2	C	27	27,0	gtG/gtC
TraesCS3D02G025600	8841406	A	G	G	3	0,3	A	33	33,0	ttT/ttC
TraesCS3D02G027500	9318603	G	C	C	32	1,31	S	2	1,1	gcG/gcC
TraesCS3D02G030600	11354815	C	G	G	3	0,3	C	4	4,0	Cag/Gag
TraesCS3D02G030600	11354829	T	C	C	3	0,3	T	4	4,0	aaT/aaC
TraesCS3D02G030600	11354975	C	T	T	6	0,6	N	.	.	gCg/gTg
TraesCS3D02G030600	11355056	A	C	C	5	0,5	A	3	3,0	gAg/gCg
TraesCS3D02G030600	11355198	C	T	T	4	0,4	C	11	11,0	taC/taT
TraesCS3D02G030600	11355529	A	G	G	2	0,2	A	3	3,0	Aac/Gac
TraesCS3D02G030600	11355673	A	G	G	3	0,3	A	2	2,0	Aat/Gat
TraesCS3D02G030800	11364323	A	G	G	3	0,3	A	12	12,0	acT/acC
TraesCS3D02G030800	11364425	G	A	A	7	0,7	G	6	6,0	tgC/tgT
TraesCS3D02G030800	11364583	G	T	T	4	0,4	G	5	5,0	cCa/cAa
TraesCS3D02G030800	11366309	T	C	C	2	0,2	T	23	23,0	Atg/Gtg

Table S4 information of gene function annotation

Gene_ID	TrEMBL_annotation	eggNOG_class_annotation	nr_annotation
TraesCS3D02G018800	Uncharacterized protein {ECO:0000313|EnsemblPlants:Traes_3B_AFA81CB35.2} OS=Triticum aestivum (Wheat) PE=4 SV=1	Intracellular trafficking, secretion, and vesicular transport	hypothetical protein F775_52397 [Aegilops tauschii]
TraesCS3D02G018900	Predicted protein {ECO:0000313|EMBL:BAK06281.1} OS=Hordeum vulgare var. distichum (Domesticated barley) PE=2 SV=1	Carbohydrate transport and metabolism	unnamed protein product [Triticum aestivum]
TraesCS3D02G019000	Uncharacterized protein {ECO:0000313|EnsemblPlants:Traes_3B_77C6EE076.1} OS=Triticum aestivum (Wheat) PE=3 SV=1	Secondary metabolites biosynthesis, transport and catabolism	Putative LRR receptor-like serine/threonine-protein kinase [Aegilops tauschii]
TraesCS3D02G019100	10-deacetylbaccatin III 10-O-acetyltransferase {ECO:0000313|EMBL:EMT23250.1} OS=Aegilops tauschii (Tausch's goatgrass) PE=4 SV=1	--	hypothetical protein F775_10541 [Aegilops tauschii]
TraesCS3D02G019200	Putative decarboxylase protein {ECO:0000313|EMBL:ADE48535.1} OS=Triticum aestivum (Wheat) PE=3 SV=1	Amino acid transport and metabolism	hypothetical protein F775_04238 [Aegilops tauschii]
TraesCS3D02G019300	Taxadien-5-alpha-ol O-acetyltransferase {ECO:0000313|EMBL:EMT23248.1} OS=Aegilops tauschii (Tausch's goatgrass) PE=4 SV=1	Function unknown	acyl transferase 15-like [Aegilops tauschii subsp. tauschii]
TraesCS3D02G019400	Uncharacterized protein {ECO:0000313|EMBL:EMT23247.1} OS=Aegilops tauschii (Tausch's goatgrass) PE=4 SV=1	Function unknown	receptor kinase [Triticum aestivum]
TraesCS3D02G019500	Chromosome 3B, genomic scaffold, cultivar Chinese Spring {ECO:0000313|EMBL:CDM80381.1} OS=Triticum aestivum (Wheat) PE=4 SV=1	Function unknown	unnamed protein product [Triticum aestivum]
TraesCS3D02G019600	Uncharacterized protein {ECO:0000313|EnsemblPlants:TRIUR3_20025-P1} OS=Triticum urartu (Red wild einkorn) PE=4 SV=1	--	Indole-3-glycerol phosphate lyase, chloroplastic [Aegilops tauschii]

TraesCS3D02G019700	Uncharacterized protein {ECO:0000313|EMBL:CCG48022.1} OS=Triticum aestivum (Wheat) PE=4 SV=1	Function unknown	hypothetical protein F775_15062 [Aegilops tauschii]
TraesCS3D02G019800	Cytochrome P450, putative {ECO:0000313|EMBL:CCG48021.1} OS=Triticum aestivum (Wheat) PE=3 SV=1	Secondary metabolites biosynthesis, transport and catabolism	E3 ubiquitin-protein ligase SINA-like 10 [Aegilops tauschii]
TraesCS3D02G019900	Zinc finger, C3HC4 type (RING finger) domain containing protein {ECO:0000313|EMBL:CCG48020.1} OS=Triticum aestivum (Wheat) PE=4 SV=1	RNA processing and modification	polygalacturonase, putative, expressed [Triticum aestivum]
TraesCS3D02G020000	Methyltransferase domain containing protein,expressed {ECO:0000313|EMBL:CCG48019.1} OS=Triticum aestivum (Wheat) PE=4 SV=1	General function prediction only	Germin-like protein 8-11 [Aegilops tauschii]
TraesCS3D02G020100	Uncharacterized protein {ECO:0000313|EMBL:CCG48018.1} OS=Triticum aestivum (Wheat) PE=4 SV=1	Function unknown	hypothetical protein F775_12851 [Aegilops tauschii]
TraesCS3D02G020200	Eukaryotic translation initiation factor 3 subunit A {ECO:0000256|HAMAP-Rule:MF_03000, ECO:0000256|SAAS:SAAS00003684} OS=Triticum aestivum (Wheat) PE=3 SV=1	Translation, ribosomal structure and biogenesis	unnamed protein product [Triticum aestivum]
TraesCS3D02G020300	Uncharacterized protein {ECO:0000313|EnsemblPlants:MLOC_26770.1} OS=Hordeum vulgare var. distichum (Domesticated barley) PE=4 SV=1	Energy production and conversion	hypothetical protein TRIUR3_02423 [Triticum urartu]
TraesCS3D02G020400	F-box domain containing protein,putative,expressed {ECO:0000313|EMBL:CCG48016.1} OS=Triticum aestivum (Wheat) PE=4 SV=1	General function prediction only	E3 ubiquitin-protein ligase SINA-like 10 [Aegilops tauschii]/F-box protein
TraesCS3D02G020500	Cystatin, expressed {ECO:0000313|EMBL:CCG48015.1} OS=Triticum aestivum (Wheat) PE=4 SV=1	General function prediction only	Putative ubiquitin carrier protein E2 23 [Aegilops tauschii]
TraesCS3D02G020600	Uncharacterized protein {ECO:0000313|EMBL:CCG48014.1} OS=Triticum aestivum (Wheat) PE=4 SV=1	General function prediction only	General transcription factor IIE subunit 2 [Aegilops tauschii]
TraesCS3D02G020700	Uncharacterized protein {ECO:0000313|EMBL:CCG48013.1} OS=Triticum aestivum (Wheat) PE=4 SV=1	General function prediction only	Putative membrane protein [Aegilops tauschii]
TraesCS3D02G020800	E3 ubiquitin-protein ligase {ECO:0000256|RuleBase:RU201113} OS=Triticum aestivum (Wheat) PE=3 SV=1	General function prediction only	unnamed protein product [Triticum aestivum]

TraesCS3D02G020900	Uncharacterized protein {ECO:0000313|EMBL:EMT24721.1} OS=Aegilops tauschii (Tausch's goatgrass) PE=4 SV=1	--	PREDICTED: ethylene-responsive proteinase inhibitor 1-like [Setaria italica]
TraesCS3D02G021000	General transcription factor IIE subunit 2 {ECO:0000313|EMBL:EMT24722.1} OS=Aegilops tauschii (Tausch's goatgrass) PE=4 SV=1	Transcription	predicted protein [Hordeum vulgare subsp. vulgare]
TraesCS3D02G021100	HCBT-like defense response protein,putative,expressed {ECO:0000313|EMBL:CCG48011.1} OS=Triticum aestivum (Wheat) PE=4 SV=1	Function unknown	hypothetical protein F775_04622 [Aegilops tauschii]
TraesCS3D02G021200	Sarcoplasmic reticulum histidine-rich calcium-binding protein, putative, expressed {ECO:0000313|EMBL:CCG48010.1} OS=Triticum aestivum (Wheat) PE=4 SV=1	Function unknown	hypothetical protein F775_15060 [Aegilops tauschii]
TraesCS3D02G021300	Terpene synthase, putative {ECO:0000313|EMBL:CCG48009.1} OS=Triticum aestivum (Wheat) PE=4 SV=1	General function prediction only	conserved hypothetical protein, expressed [Triticum aestivum]
TraesCS3D02G021400	Uncharacterized protein {ECO:0000313|EnsemblPlants:TRIUR3_01778-P1} OS=Triticum urartu (Red wild einkorn) PE=4 SV=1	Transcription	Aspartic proteinase nepenthesin-2 [Aegilops tauschii]
TraesCS3D02G021500	Uncharacterized protein {ECO:0000313|EMBL:EMT04100.1} OS=Aegilops tauschii (Tausch's goatgrass) PE=4 SV=1	Function unknown	Myb-related protein 305 [Aegilops tauschii]
TraesCS3D02G021600	Terpene synthase, putative, expressed {ECO:0000313|EMBL:CCG48008.1} OS=Triticum aestivum (Wheat) PE=4 SV=1	General function prediction only	Beta-glucosidase 44 [Aegilops tauschii]
TraesCS3D02G021700	Polygalacturonase, putative, expressed {ECO:0000313|EMBL:CCG48006.1} OS=Triticum aestivum (Wheat) PE=3 SV=1	General function prediction only	Subtilisin-like protease [Aegilops tauschii]
TraesCS3D02G021800	tRNA (Guanine-N(1)-)-methyltransferase,putative,expressed {ECO:0000313|EMBL:CCG48005.1} OS=Triticum aestivum (Wheat) PE=4 SV=1	Translation, ribosomal structure and biogenesis	hypothetical protein TRIUR3_14872 [Triticum urartu]
TraesCS3D02G021900	Putative disease resistance protein {ECO:0000313|EMBL:EMT11538.1} OS=Aegilops tauschii (Tausch's goatgrass) PE=4 SV=1	Signal transduction mechanisms	unnamed protein product [Triticum aestivum]
TraesCS3D02G022000	Uncharacterized protein {ECO:0000313|EMBL:CCG48004.1} OS=Triticum aestivum (Wheat) PE=4 SV=1	Function unknown	predicted protein [Hordeum vulgare subsp. vulgare]
TraesCS3D02G022100	Uncharacterized protein {ECO:0000313|EMBL:CCG48003.1} OS=Triticum aestivum (Wheat) PE=4 SV=1	Function unknown	terpene synthase, putative, expressed [Triticum aestivum]

TraesCS3D02G022200	Alanyl-tRNA synthetase, putative, expressed {ECO:0000313|EMBL:CCG48002.1} OS=Triticum aestivum (Wheat) PE=4 SV=1	General function prediction only	xyloglucan galactosyltransferase KATAMARI 1,putative, expressed [Triticum aestivum]
TraesCS3D02G022300	Exonuclease, putative {ECO:0000313|EMBL:CCG48001.1} OS=Triticum aestivum (Wheat) PE=4 SV=1	Replication, recombination and repair	conserved hypothetical protein, expressed [Triticum aestivum]
TraesCS3D02G022400	Leucyl-tRNA synthetase, putative, expressed {ECO:0000313|EMBL:CCG48000.1} OS=Triticum aestivum (Wheat) PE=3 SV=1	Translation, ribosomal structure and biogenesis	Putative acetyl-CoA acetyltransferase, cytosolic 2 [Aegilops tauschii]
TraesCS3D02G022500	Glycosyltransferase, HGA-like,putative,expressed {ECO:0000313|EMBL:CCG47999.1} OS=Triticum aestivum (Wheat) PE=4 SV=1	Function unknown	unnamed protein product [Triticum aestivum]
TraesCS3D02G022600	Coatomer subunit beta'-1 {ECO:0000313|EMBL:EMT11531.1} OS=Aegilops tauschii (Tausch's goatgrass) PE=4 SV=1	Signal transduction mechanisms	unnamed protein product [Triticum aestivum]
TraesCS3D02G022700	Chromosome 3B, genomic scaffold, cultivar Chinese Spring {ECO:0000313|EMBL:CDM80417.1} OS=Triticum aestivum (Wheat) PE=4 SV=1	--	Disease resistance protein RPM1 [Aegilops tauschii]
TraesCS3D02G022800	Uncharacterized protein {ECO:0000313|EnsemblPlants:Traes_3B_663315A98.2} OS=Triticum aestivum (Wheat) PE=4 SV=1	--	unnamed protein product [Triticum aestivum]
TraesCS3D01G022900	Salt overly sensitive 1 {ECO:0000313|EMBL:AIA08675.1} OS=Triticum aestivum (Wheat) PE=2 SV=1	Inorganic ion transport and metabolism	plasma membrane Na+/H+ antiporter [Triticum aestivum]
